# From Wish to Reality: Soteria in Regular Care—Proof of Effectiveness of the Implementation of Soteria Elements in Acute Psychiatry

**DOI:** 10.3389/fpsyt.2021.685779

**Published:** 2021-07-09

**Authors:** Theresa Wolf, Philine Fabel, Adrian Kraschewski, Maria C. Jockers-Scherübl

**Affiliations:** Department of Psychiatry and Psychotherapy, Academic Teaching Hospital of the Charité Berlin, Oberhavel Kliniken GmbH, Hennigsdorf, Germany

**Keywords:** soteria, coercive measures, acute psychiatric care, inpatient treatment, locked ward

## Abstract

**Objective:** This article examines the influence of the implementation of Soteria elements on coercive measures in an acute psychiatric ward after reconstruction in 2017, thereby comparing the year 2016 to the year 2019. The special feature is that this is the only acute psychiatric ward in Hennigsdorf Hospital, connected now both spatially and therapeutically to an open ward and focusing on the treatment of patients suffering from schizophrenia and schizophrenia spectrum disorders.

**Methods:** The following parameters were examined: aggressive assaults, use of coercion (mechanical restraints), duration of treatment in open or locked ward, type of discharge, coercive medication, and dosage of applied antipsychotics. For this purpose, the data of all legally accommodated patients in the year 2016 (before the reconstruction) and 2019 (after the reconstruction) were statistically analyzed in a pre–post mirror quasi-experimental design.

**Results:** In 2019, the criteria of the Soteria Fidelity Scale for a ward with Soteria elements were reached. In comparison to 2016 with a comparable care situation and a comparable patient clientele, there was now a significant decrease in aggressive behavior toward staff and fellow patients, a significantly reduced number of fixations, a significantly reduced overall duration of inpatient stay, and a significant increase in treatment time in the open area of our acute ward.

**Conclusion:** The establishment of Soteria elements in the acute psychiatric ward leads to a verifiable less violent environment of care for severely ill patients and to a drastic reduction in coercive measures.

## Introduction

Since more than 100 years, there were efforts in psychiatry to reduce coercive measures with the goal not only to treat inpatients with respect but also to protect those working there and thus avoid a “brutalization” of the staff (1). In the 1970s in the United States, Mosher developed the so-called Soteria concept. Soteria literally means salvation or deliverance in Greek. Initially, Mosher established Soteria as an anti-psychiatric approach with primarily “laymen” as milieu therapists, who, if possible, had never worked in psychiatry before (2). In the tradition of Pinel (1797) as a “traitement moral,” they should definitely feel obliged to a humanistic approach (3). The concept was aiming at patients with schizophrenia and schizophrenia spectrum disorder. Similarly, in Europe, there was the Psychiatry Enquête advocating de-hospitalization and strengthening patients' rights. In Berne, Switzerland, the Soteria treatment was established by Ciompi who emphasized the relaxing, neuroleptic-like effect of a less irritating environment close to everyday life (4). Its clinical effectiveness has been proven many times (5, 6). However, data on the clinical effectiveness and efficacy of Soteria were only explicitly analyzed by the working groups around Mosher and Ciompi, showing equal or better outcomes of Soteria treatment compared to regular treatment. The former could statistically prove that, over a 2-year period, those in the intervention group compared to those in the control group (antipsychotics as usual in an inpatient ward) are more likely to live alone or with peers, without differences in re-admission, symptoms, social function, or employment. A subsample of patients diagnosed with schizophrenia had better and more improved global psychopathology and better social outcomes, including 40% higher probability of employment (3). For the Soteria Berne, at 2 years, the intervention and the control group did not show any statistical difference for relapse, symptoms, or function (7, 8). Both studies needed significantly less antipsychotics for their intervention groups. Mosher et al. (9) reported that, after 1 year in the Soteria USA, 10% in the intervention group and 75–100% in the control group received antipsychotics. For the Soteria Berne, the total dosage of medication was less than half for residents in the Soteria compared to the control group (8). Thus, keeping in mind the usual dose of antipsychotics at that time [e.g., 700 mg/day chlorpromazine equivalents (CPZ) (3)], their known side effects, and likewise the also well-known non-compliance of 42–50% of patients (10), an important therapeutic alternative had been established with the Soteria approach.

In German-speaking countries, the Soteria concept was implemented in some hospitals in open wards (München Ost KBO Isar-Amper- Klinikum, Berlin St. Hedwig, LVR Klinik Bonn, Zentrum für Psychiatrie Rheinau, Vianobis Fachklinik Gangelt, Münsterklinik Zwiefalten). In the LWL Klinik Gütersloh, under the chief medical direction of Professor Klaus Dörner, Soteria elements were also established in the acute psychiatric ward, showing a reduction in coercive and violent measures. They also report an improvement in the ward atmosphere for patients and staff (11), however lacking empirical analysis. Unfortunately, the project could not be continued after a change of staff and currently does not meet the criteria of the Soteria Fidelity Scale.

In addition, there have been continuing efforts to reduce coercive measures in the acute wards of psychiatric hospitals. Last but not least, the ratification of the UN Convention on the Rights of Persons with Disabilities (12), which originally intended to abolish coercive measures completely, led to a more critical discussion of coercive measures in Germany (13). In the course of this, the Legal Guardian Law and the mental health law [formal detention initiated by the patients' legal guardians (BGB), compulsory detention by Federal Land Laws (BbgPsychKG), and Forensic Psychiatry Laws] were revised toward a more restricted use of coercive measures within psychiatry and forensics (13). Two approaches are emerging in German-speaking^1^ countries. On the one hand, efforts that aim to significantly change the milieu of a locked ward, e.g., the Safewards project (15) at the Urban Klinikum Berlin Kreuzberg (16) or the “open door” project at the Charité Berlin Mitte (17) should be mentioned. At the Urban Klinikum Berlin Kreuzberg, an overall reduction of coercive measures and their duration as well as a reduced necessity of compulsory medication could be demonstrated after implementation (16). At the Hospital Charité Berlin Mitte, the open door policy led to a reduced administration of coercive medication and an overall decrease in aggressive assaults without an increase in therapy discontinuations or number of fixations nor special safety measures (17). Another approach aims at improving the care situation by integrating more closely the inpatient and outpatient sectors. The efforts have been successfully implemented since 2012 through a change in the law and the resulting possibility of an annual budget for hospitals in Germany. In the application, not only a reduction in the length of treatment in hospitals but also a reduction in sick leave overall as well as improved acceptance by clients and staff could be achieved (18, 19). By reducing the number of involuntary treatments, the model of Integrated Care in Hamburg was able to reduce coercive measures indirectly (20, 21). With the Weddinger Model in Berlin, a combined approach can be found, combining outpatient and inpatient treatment on the one hand and an additional change in the acute psychiatric setting on the other hand, including opening of ward doors, debriefing of coercive measures, and inclusion of peers (22). In consequence, a reduction of involuntary treatment, total treatment time, and number of fixations could be demonstrated directly in the inpatient setting (23).

In 2017, our acute psychiatric ward was reconstructed and, in order to reduce coercive measures, in the following year we tried to establish an alternative way to treat our acutely ill patients by introducing Soteria elements. We are responsible for the psychiatric treatment of the Oberhavel (Brandenburg) catchment area in Germany and can thus compare the same patient clientele before and after the reconstruction and implementation of Soteria elements.

To the best of our knowledge, we are at that time the only hospital in Europe to try to establish Soteria elements in the only existing acute psychiatric inpatient unit of the hospital. With the structural change to divide the acute locked ward into a large open and a small, locked area, spatially and therapeutically connected, experiences of the Soteria concept are linked with those of the open door projects. In contrast to open door, Soteria wards, or a classic Soteria setting though, we have created a setting whereby patients requiring acute psychiatric treatment are cared for by the same team and on the same ward as those now less acute. We were thus able to provide all patients suffering from schizophrenia or schizophrenia spectrum disorder individually need-adapted with elements of the Soteria treatment, regardless of the severity of the present state. The effects of the complex intervention carried out on the frequency of special incidents, coercive measures, treatment duration as well as the level of neuroleptic dosage and frequency of the given coercive medication are the goal of our study presented here.

## Methods

### Description of the Setting

The principal idea was to change an acute psychiatric ward with 23 beds, optionally closed, to an open acute ward with Soteria elements (15 beds) and, additionally, a small protected area with six beds [corresponding to the requirement of the Psychiatry-Enquête of 1975: the size of an acute psychiatric ward should not exceed 16 beds (24)]. Both wards are structurally connected, such that patients can be cared for as needed without a change of treatment team members. Soteria means the creation of a “small, community-like, intensive, and interpersonally focused therapeutic milieu” (25). The patient is accompanied and supported in developing a way of dealing with the psychosis and to find meaning in the subjective experience. Drug treatment is carried out individually and negotiated in an open dialogue. At the same time, a crisis intervention ward has been created in the hospital with a focus on patients with a borderline personality disorder and/or acute crises in order to align the acute psychiatric ward to be more disorder-specific and thus match better to various disorder patterns in the sense of the respective guidelines.

The Oberhavel catchment area is located in the north of the greater Berlin area in the federal land of Brandenburg and has a population of about 202,000. The Department of Psychiatry and Psychotherapy of the Oberhavel Hospitals, with 101 beds and 57 day clinic places at the locations such as Hennigsdorf, Oranienburg, and Gransee as well as a large outpatient clinic, is responsible for the psychiatric treatment in the Oberhavel county. The overall aim of the hospital is to work according to a disorder-specific group therapy concept. In addition to the acute ward with Soteria elements and the crisis intervention ward, we also have an interdisciplinary geriatric–gerontopsychiatric ward, a ward for patients with affective disorders and a ward for addiction and comorbid disorders. The disorder-specific organization of our department leads to a desired focus on psychotic disorders in our acute ward and allows this ward to be kept small. The acute care unit continues to provide care for patients in the Oberhavel catchment area who are detained according to state law or according to legal guardian law in the case of reduced ability to have insight and control and for patients who voluntarily seek inpatient treatment with an acute psychosis.

### Evaluation of the Implementation

The opening of the acute care ward with Soteria elements took place in June 2018 (a detailed description of the concept and its implementation is reported elsewhere). The process was monitored in weekly, multi-professional working group meetings. The team members were given various internal and external training opportunities, and the team was externally supervised once a month. During the entire period from 2016 to 2019 (and beyond), the senior staff of the ward and the hospital did not change. In addition, there was no change in the organization of our other inpatient units or the care provided by our day-care treatment places and our outpatient services. The above-mentioned Soteria facilities provided additional support through professional exchange. The Soteria Fidelity Scale was used to evaluate the implementation, on which recognition by the International Working Group Soteria (IAS) is based (26). In the Soteria Fidelity Scale, the following areas are defined: “spatial setting” (e.g., number of beds, availability of an open ward), “care team” (e.g., inclusion of all team members, non-occupational group-specific work, proportion of working time spent on the patient), “treatment setting” (e.g., use of coercion, neuroleptic dosage, stimulus protection, relapse prevention, aftercare, and inclusion of the patient and his/her relatives), and “Soteria everyday life” (e.g., joint coping with everyday life, joint cooking). The self-rating questionnaire is to be filled out individually, and the resulting values are being averaged. The total score can then be classified into “clinical ward” (30–50P), “ward with Soteria elements” (51–70P.), and “Soteria” (71–90P.).

The following variables of all legally accommodated patients in the years 2016 (t0, before the reconstruction) and 2019 (t1, after the reconstruction) were analyzed in a pre–post mirror quasi-experimental design: special incidents^2^ reported to the Ministry of Health, number of escapes, number of re-admissions within 1 year (“revolving door effect”), use of coercive measures (mechanical restraints), application of compulsory medication in acute cases, court-approved continuous medication, duration of hospitalization^3^, duration of time in the open ward^4^, type of discharge (planned/unplanned), and neuroleptic dosage measured *via* CPZ, the determination of which was based on Benkert and Hippius (27).

Data collection was based on the compulsory annual reports to the Ministry of Health in Brandenburg on patients' legally accommodated and on special events. The data were supplemented and expanded by the letters of discharge and, since the introduction of the electronic patient file in January 2019, the electronic records on the medical order of coercive measures were added. The discharge medication documented in the discharge letter was used to analyze the CPZ. As CPZ levels can be influenced by comorbid substance use disorder (28, 29), we controlled for this and additionally compared both groups, i.e., patients with a psychotic crisis and comorbid substance use disorder and patients with a psychotic crisis without comorbid substance use disorder. The discharge letter also provided information on the exact circumstances of discharge. The data quality can therefore be rated as high. As the data processing was carried out anonymously, no approval was obtained from the ethics committee. All patients who were legally accommodated in the years 2016 and 2019 were included in the analysis.

The statistical evaluation was done with the programs SPSS 22.0 and Microsoft Excel. The target parameters were evaluated regarding differences in the groups (treatment in 2016 vs. treatment in 2019). Since the CPZ variable was not normally distributed, the Kruskal–Wallis test was used for group comparison. The chi-square test was used to calculate the frequency differences of nominally scaled variables. For metric variables, we used uni- or multivariate analysis of variance with Bonferroni adjustment for multiple testing.

## Results

The criteria according to the Soteria Fidelity Scale (26) as “ward with Soteria elements” (51–70) were met in June 2018 (with an average score of 55 p.) and in November 2019 (57 p.). The implementation was thus successful. The acknowledgment by the International Working Group Soteria (IAS) took place in December 2019 (https://soteria-netzwerk.de/soteria-einrichtungen).

### Description of the Sample

Table 1 shows the sociodemographic data, the frequency of legal accommodation [according to formal detention initiated by the patients' legal guardians (BGB) or compulsory detention by Federal Land Laws (BbgPsychKG)], and the long-term involuntary hospital treatment as well as the diagnostic distributions of the two samples. There were no significant differences in the sociodemographic parameters between 2016 and 2019. Amounting to 65%, the proportion of psychosis patients and psychosis patients with comorbid substance use disorder is the most frequent diagnostic group found in detained patients. In 2019, no patient with complex post-traumatic stress disorder was in involuntary acute care. It may be assumed that the disorder-specific offer of our crisis ward was able to avoid such an escalation. There were no significant differences between 2016 and 2019 regarding the diagnoses treated [χ^2^_(5/97)_ = 6.91, *p* = 0.228). For the years 2016 and 2019, the care situation and the characteristics of the patients in the catchment area can generally be considered comparable.

**Table 1:** Sample characteristics.

	**2016**	**2019**
Sample size (*N*)	45	52
Gender (m/f) (*n*)	28/17	28/24
Age, *M* (±SD)	41.87 (±16.24)	46.13 (±15.98)
**Accommodations (*****n*****)**		
24-h detention according to BbgPsychKG (§12, §14)	2	3
Detention according to BbgPsychKG (§8)	9	15
Detention according to BGB (§1,906)	29	30
Long-term detention according to BGB (§1,906)	5	4
**Diagnostic distributions (*****n*****)**		
Organic psychiatric diseases (F0)	6	6
Addiction disorders (F1)	4	8
Schizophrenia and bipolar psychosis (F2 and F3)	17	24
Major depressive disorder (F3)	4	2
Complex post-traumatic stress disorder	4	0
Psychosis and addiction (dual diagnosis)	10	12

*n, number of subjects; M, mean; SD, standard deviation*.

### Special Incidents

For the years 2016 and 2019, a total of 24 special incidents were reported to the Ministry of Health, all of which concerned patients with involuntary treatment. In 2016, no unauthorized leaving and no destruction of furniture were reported; however, 13 incidents of physical assault were reported. In 2019, four incidents of unauthorized leaving, four incidents of physical assault, and three incidents of destruction of furniture were reported (see Table 2). The analysis shows significantly less attacks on staff and other patients in 2019 compared to 2016 [χ^2^_(2/24)_ = 11.68, *p* = 0.003].

**Table 2:** Number of special incidents, treatment duration, number of re-admissions, discharge circumstances, coercive measures, and chlorpromazine equivalents before and after the implementation of Soteria elements.

	**2016**	**2019**	**Statistics**
Sample size (*N*)	45	52	
**Special incidents (*****n*****)**			
Type of incident			χ^2^_(2/24)_ = 11.68, *p* = 0.003^**^
Unauthorized leaving	0	4	
Assault	13	4	
Destruction of furniture	0	3	
Severity of assault			χ^2^_(2/17)_ = 2.55, *p* = 0.279
Slight	1	1	
Moderate	7	3	
Severe	5	0	
**Treatment duration in days (*****M*** **±** **SD)**			
Maximum allowed legal detention time (by law)	95.16 (±120.19)	67.31 (±89.53)	*F*_(1/97)_ = 1.70, *p* = 0.195
Actual detention time	67.29 (±90.11)	42.58 (±63.26)	*F*_(1/97)_ = 2.49, *p* = 0.118
Total length of stay per year	56.87 (±47.81)	39.31 (±28.92)	*F*_(1/97)_ = 4.93, *p* = 0.029^*^
Treatment time in an open ward	2.33 (±9.03)	13.15 (±22.88)	*F*_(1/97)_ = 8.86, *p* = 0.004^**^
Voluntary follow-up treatment	9.84 (±18.64)	11.25 (±20.06)	*F*_(1/97)_ = 0.13, *p* = 0.72
**Re-admissions**			
Number of re-admissions per patient (*M* ± SD)	1.71 (±0.94)	1.65 (±1.57)	*F*_(1/97)_ = 0.46, *p* = 0.83
Patients (*n*) with re-admissions (yes/no)	22/23	16/36	*χ^2^*_(1/97)_ = 3.32, *p* = 0.068
**Discharge circumstances (*****n*****)**			
planned (yes/no)	35/10	47/5	*χ^2^*_(1/97)_ = 2.93, *p* = 0.87
**Coercive measures**			
Administration of acute forced medication (*M* ± SD)	0.51 (±1.18)	0.37 (±0.63)	*F*_(1/97)_ = 599, *p* = 0.441
Number of fixations (*M* ± SD)	4.53 (±10.60)	0.81 (±1.59)	*F*_(1/97)_ = 6.27, *p* = 0.014^*^
Administration of acute forced medication (yes/no)	14/31	15/37	χ^2^_(1/97)_ = 0.059, *p* = 0.81
Court-approved continuous medication (yes/no)	6/39	6/46	χ^2^_(1/97)_ = 0.72, *p* = 0.79
**Medication**			
*N*	30	32	
CPZ values	441.65 (±322.18), rank 32	533.81 (±466.52), rank 33.86	KS value: *χ^2^*_(1/65)_ = 1.57, *p* = 0.692

*n, number of subjects; M, mean value; SD, standard deviation*.

* *p < 0.05;*

** *p < 0.01;*

*** p < 0.001.

The difference in severity of assaults misses statistical significance [χ^2^_(2/17)_ = 2.55, *p* = 0.279] since the number of reported cases is low. Nevertheless, not a single case of serious assault was reported in 2019.

### Treatment Duration

The comparison of frequencies of hospitalization and detention time was analyzed. Between the years 2016 and 2019, there was no significant difference in the approved [*F*_(1/97)_ = 1.70, *p* = 0.195] or actual [*F*_(1/97)_ = 2.49, *p* = 0.118] involuntary accommodation time. Due to the large scattering, the differences in mean values are not significant. For 2019, however, a slight reduction is seen (see Table 2). The comparison of treatment time in days shows statistical relevant differences. The treatment time in days had significantly decreased [*F*_(1/97)_ = 4.93, *p* = 0.029] just as the treatment time in the open area had significantly increased [*F*_(1/97)_ = 8.86, *p* = 0.004]. Nevertheless, there was no difference in the number of days that the patients decided to continue treatment voluntarily [*F*_(1/97)_ = 0.13, *p* = 0.72]. The exclusion of patients with long-term involuntary accommodation did not also result in any significant difference in values or statistics and is therefore not shown.

### Re-admissions

There was no significant difference in the number of multiple hospitalizations of an involuntarily accommodated patient [*F*_(1/97)_ = 0.46, *p* = 0.83; see Table 2). However, the proportion of patients admitted multiple times in 2016 tended to be higher than in 2019, missing statistical significance though [χ^2^_(1/97)_ = 3.32, *p* = 0.068]. The exclusion of the patients with long-term involuntary hospital treatments according to BGB (365 days, *n* = 9 in total) showed no difference in outcome and is therefore not reported.

### Discharge Circumstances

Comparing 2016 and 2019, the relation between planned discharge and premature discontinuation [χ^2^_(1/97)_ = 2.93, *p* = 0.087] was not statistically significant. This means that, in spite of the possibility of a treatment in the open ward for accommodated patients in 2019, there was no increase in the number of unauthorized leavings.

### Coercive Measures

The frequency of administered acute forced medication, the frequency of court-approved continuous medication, and the number of mechanical fixations were analyzed (see Table 2). Between the years 2016 and 2019, neither the frequency of administered acute medication in an emergency situation [*n* = 14 vs. *n* = 15; χ^2^_(1/97)_ = 0.059, *p* = 0.81] nor the frequency of court-approved continuous medication [*n* = 6 vs. *n* = 6, χ^2^_(1/97)_ = 0.72, *p* = 0.79] changed.

**Figure 1:**
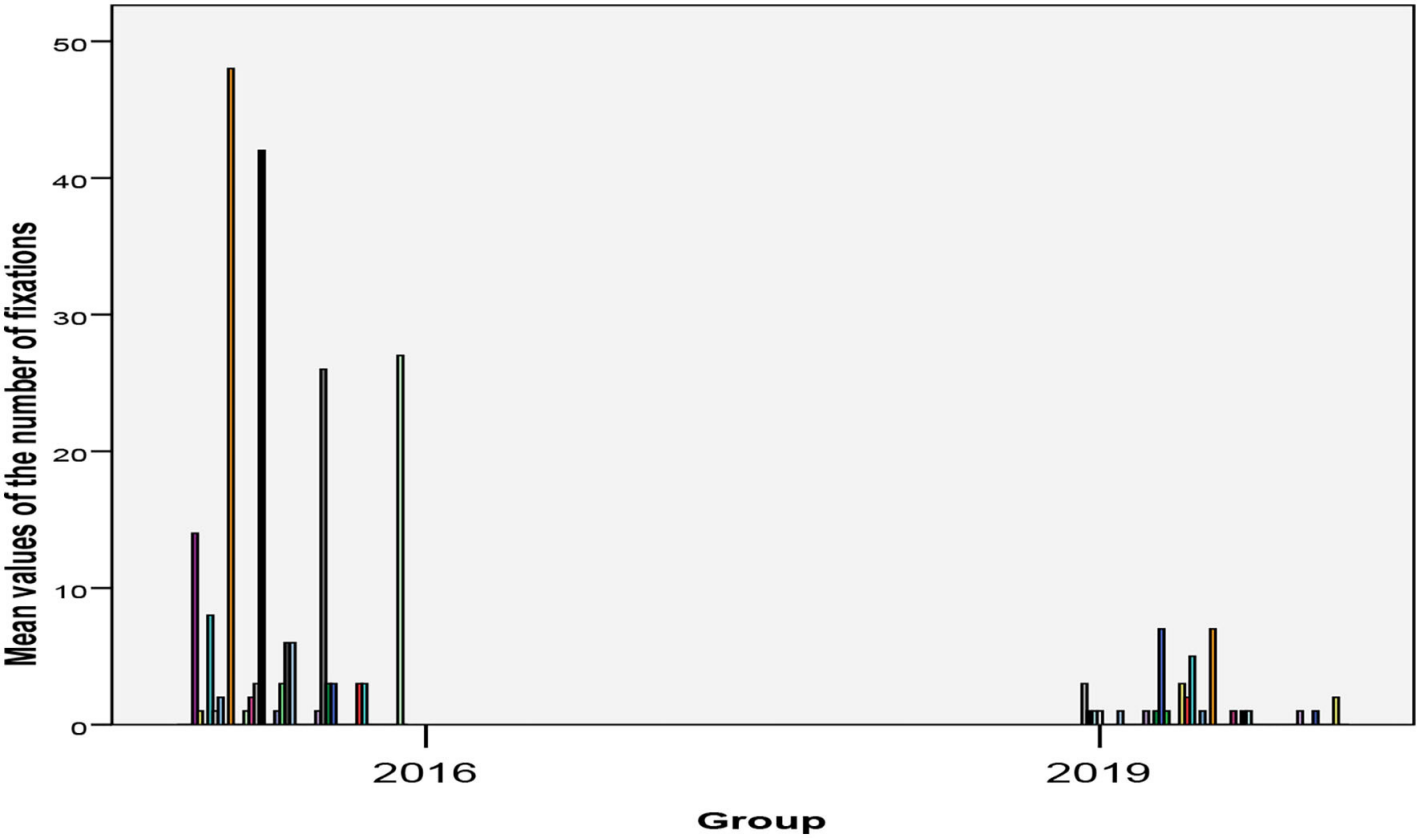
Frequency of mechanical fixations per affected patient separated by years.

As Figure 1 shows, in 2016, 21 of 45 (48.8%) involuntarily accommodated patients were mechanically restrained during their stay, with a frequency range of once or twice (seven cases) up to 10 times (two cases), over 20 times (two cases), and up to over 40 times (also two cases). In 2019, however, only 20 of 52 (38.5%) patients were mechanically restrained during their stay, with a maximum of seven times (two cases). The majority (13 cases) only had to be mechanically restrained once during their stay. There was a dramatic decrease in the frequency of mechanical restraints [*F*_(1/97)_ = 6.27, *p* = 0.014, see Table 2].

### Medication

In 2016, 12 of 45 involuntarily accommodated patients were discharged without psychopharmacological medication. In 2019, 11 of 52 were discharged without medication; this difference is not statistically significant [χ^2^_(1/97)_ = 0.405, *p* = 0.524]. Out of the resulting 74 patients receiving psychopharmacological medication, 65 patients were receiving antipsychotic medication. We evaluated the CPZ for those 65 patients (see Table 2). Since these were not normally distributed, we used the Kruskal–Wallis test in addition to the single-factor analysis of variance. Comparing the 2 years, the dosage of the neuroleptics is statistically equal [χ^2^_(1/65)_ = 1.57, *p* = 0.692]. Even for the group of patients with a psychotic crisis with or without additional substance use disorder (*N* = 63), we could not find a CPZ change [χ^2^_(1/63)_ = 0.024, *p* = 0.878]. Similarly, we found no significant difference in CPZ levels between patients with additional substance use disorder and patients with a psychotic crisis solely [χ^2^_(1/63)_ = 2.64, *p* = 0.104]. Statistical significance was missed here in both 2016 and 2019 and is therefore not reported.

## Discussion

We could show that the treatment on an acute psychiatric ward with Soteria elements compared to traditional treatment in a comparable care situation and patient clientele leads to significantly less aggressive assaults on staff and other patients, significantly reduced overall length of stay, significantly longer treatment time in open ward, and significantly less fixations. The severity of incidents and the number of re-admissions were decreasing, although missing statistical significance. In 2019, it was possible to treat patients with involuntary accommodation in the open area of our acute ward more quickly without changing wards or teams while at the same time reducing the overall treatment time for patients. This is all the more pleasing because, as reported, neither the discontinuation rates nor the number of readmissions (returning patients or re-admissions, respectively) had increased as a result. On the contrary, there was rather a tendency toward a decrease in patient readmissions in 2019. There were no relevant differences in medication at the time of discharge. Hence, it may be assumed that the severity of a disease leading to hospital detention required the same drug treatment in both 2016 and 2019.

As the revision of the Soteria Fidelity Scale (26) emphasizes, a Soteria *per se* is only conceivable without the use of coercive measures. Nevertheless, in the present study, we could show for the first time that the linking of a ward with Soteria elements can have direct effects on an acute ward with a legal care mandate. It should be pointed out again that this is the only acute psychiatric ward in the catchment area and has to fit all patients there. An assignment of patients to an alternative locked ward is not possible, neither in the district nor in our hospital. In contrast to other studies recently investigating the impact of model projects in German-speaking countries on the sector of acute psychiatric treatment (21, 23), our present analysis only included the data of legally institutionalized patients, i.e., not the data of all patients treated in 2016 and 2019 (those that were analyzed). We could also replicate the experiences and results of implementation of Soteria elements in an acute ward published by Kroll (11).

We assume that the number of unreported special incidents will not be completely clarified due to the retrospective data collection. Nevertheless, we consider that the reporting behavior of the responsible nursing staff on duty did not differ systematically between 2016 and 2019. In Brandenburg, it is mandatory to report annually any incidents involving legally admitted psychiatric patients to the Ministry of Health. This was the same procedure in 2016 and in 2019 and includes any aggressive assault on either staff or other patients since 2011. Additionally, our psychiatric team reports any incident leading to the destruction of furnishing or inventory since 2010. In the present study as well as in the study of the Charité Berlin Mitte, a decrease in aggressive incidents could be shown, which implies that the figures of our study are quite comparable to those of Charité Berlin Mitte.

The colleagues at the Charité were not able to demonstrate a reduction of mechanical restraints due to the implemented ward policy. However, they point out a significantly lower incidence of fixations (23.3% of all institutionalized patients) compared to the Berlin average [40.4% of all institutionalized patients; unpublished data of the Berlin Senate Administration (17)]. In Hennigsdorf, the proportion of 46.7% in 2016 decreased to 38.5% in 2019, which is close to the Berlin average. If fixation was required at all for a certain patient in our study, it did never exceed the number of seven times in 2019, whereas in 2016 the maximum was 40 times in a certain patient. Thus, the implementation of a model project in order to reduce coercive measures seems to meet the requirements of the UN Convention on the Rights of Persons with Disabilities [for similar effects, see also (16, 17, 23)].

We were not able to prove a reduction in the application of acute forced medication or the frequency of the court-approved continuous medication, contrary to our expectations and the results of comparable model projects. For example, Charité Berlin Mitte could show a reduction of coercive medication, but not of mechanical restraints (17). The working group of the urban hospital showed a reduction in medication and mechanical restraints, but in relation to sample size and selected period of investigation, the frequency still appears high (16): 35 of 49 patients within 11 weeks (Urban Klinikum) vs. 20 of 52 patients within 52 weeks (Oberhavel Klinik Hennigsdorf). At St. Hedwig Hospital, Wullschleger et al. (23) did not report the variable forced medication. In summary, it seems that, regardless of the time period, a comparatively low incidence of compulsory medication necessitates a higher incidence of mechanical fixation. We are, of course, aware that comparability between hospitals can only ever be limited, as it depends on many other factors such as socio-demographic differences of the catchment area, bed occupancy of preconnected emergency ambulance with the possibility for alcohol or drug detoxication, dose of psychotropic drugs administered (measured *via* CPZ), bed occupancy of the acute ward, number of staff on duty, professional experience of staff, and attempts at de-escalation prior to implementation of restraint (30, 31). Health services research, even with a quasi-experimental design, is still field research. Perhaps it is simply important to note that the respective hospital staff succeeded in reducing coercive measures compared to the period before the intervention.

The level of prescribed neuroleptics measured *via* CPZ, a core criterion of the Soteria idea, also remained comparable. This is probably due to the fact that, unlike the working groups around Mosher and Ciompi, we examined patients with a corresponding degree of severity of the disease, who therefore require a higher neuroleptic dosage for recovery. Our data are in line with those of a Norwegian National Health study of acute psychiatric patients (32) (CPZ: MW = 450). We were able to exclude additional substance abuse as a moderating factor. Previous studies (28, 29) showed a mitigating effect of comorbid substance use in psychotic patients on CPZ dosages. Nevertheless, this effect disappeared when controlling for sociodemographic data and length of stay. Therefore, it is conceivable that the effect also loses impact with a certain degree of severity of the disease. It has to be taken into account that previous studies (28, 29) included all patients of a hospital, while the present study focuses exclusively on legally accommodated and thus more severely affected patients.

As demonstrated in comparable open door projects (33), the increase in treatment time in the open sector does not lead to an increase in escapes nor to more treatment discontinuations in our study. Additionally, the specific design of our ward environment with Soteria elements, the disorder-specific orientation, and even more so the therapeutic attitude seem to contribute to the de-escalation. In line with that, the results show a reduced number of fixations and, by decreasing the number of special incidents, also a shortened overall length of treatment—even when the patients' ability to self-control and rational thinking (the basis of compulsory institutionalization in Germany) is limited. This contradicts all prejudices regarding the Soteria (34). Furthermore, it is conceivable that a shortened treatment time, which could be achieved in the present study, may not only result in health economic cost reductions but also in the promotion of social integration and a counteraction to hospitalization (35, 36). This may have drastic short- and long-term effects on the quality of the patients' life.

The Soteria idea is approaching its 50th birthday and, in contrast to many prejudices, it still has the potential to meet the demands of modern acute care. To achieve this, the authors Schöttle and Gallinat (37) claim, among other things, sufficient staffing, a reduction in coercive measures, and a therapy ward, instead of a classical acute locked ward, that works according to a “recovery”-oriented view are indispensable.

## Limitations

A causal interpretation is not permitted in the pre–post mirror quasi-experimental design presented here. The data acquisition was retrospective and is therefore limited to the variables mentioned, although high data quality is guaranteed. In the selected period, a new remuneration system (PEPP) was introduced (01.01.2019), which may also have had an effect on the shortening of the length of stay. The extended length of stay in the open area of our acute ward will probably not have been affected by this and can therefore be associated with the implementation of Soteria elements.

## Data Availability Statement

The raw data supporting the conclusions of this article will be made available by the authors, without undue reservation.

## Ethics Statement

Since the analysis of the data did not imply the direct involvement of patients, an ethics approval for the study was not required. The Oberhavel Klinik Hennigsdorf is obligated to report all cases of coercive measures to the Ministry of Health in Brandenburg/Germany annually. We exclusively analyzed data extracted from those annual reports and completed the data with anonymized information from the hospital information system.

## Author Contributions

All the authors listed have made a substantial, direct and intellectual contribution to this work, and approved it for publication.

## Conflict of Interest

The authors declare that the research was conducted in the absence of any commercial or financial relationships that could be construed as a potential conflict of interest.

## Footnotes

^1^In the following, the focus will be on model projects in German-speaking countries since the legal provisions for treatment in case of self-endangerment and extraneous endangerment or for curative treatment are comparable [cf. differences in the use of coercive measures in Europe (14)].

^2^This includes completed suicides and suicide attempts and assaults on employees and fellow patients. In both years, there was neither a completed suicide nor an attempted suicide in the acute psychiatric ward. The gradations of assaults (mild, moderate, and severe) are defined as follows: mild: pinching, pulling, pushing, holding back, standing on the ground, and spitting; moderate: slapping, beating, kicking, boxing, holding, throwing to the ground, biting, grabbing, tearing hair, scratching, shaking, and bumping; and severe: choking, physical sexual harassment, any attack that causes physically harm to the person attacked, and any attack that is carried out with an object. The definition is noted on the registration form and is therefore accessible to every caregiver.

^3^The period between January 1 and December 31 of the years compared was counted for both years 2016 and 2019.

^4^The period between January 1 and December 31 of the years compared was counted for both years 2016 and 2019. In 2016, treatment time in the open area meant a transfer to another open ward in the hospital. This also meant a change of team.
